# Regulation of fruit ripening by the brassinosteroid biosynthetic gene *SlCYP90B3* via an ethylene-dependent pathway in tomato

**DOI:** 10.1038/s41438-020-00383-0

**Published:** 2020-10-01

**Authors:** Songshen Hu, Lihong Liu, Shuo Li, Zhiyong Shao, Fanliang Meng, Haoran Liu, Wenyi Duan, Dongyi Liang, Changqing Zhu, Tao Xu, Qiaomei Wang

**Affiliations:** 1 State Agricultural Ministry Laboratory of Horticultural Crop Growth and Development, Department of Horticulture, Zhejiang University, Hangzhou, 310058 China; 2 Key Laboratory of Protected Horticulture of Ministry of Education, College of Horticulture, Shenyang Agricultural University, Shenyang, 110866 China

**Keywords:** Metabolic engineering, Molecular engineering in plants

## Abstract

The essential role of ethylene in fruit ripening has been thoroughly studied. However, the involvement of brassinosteroids (BRs) in the regulation of fruit ripening and their relationship with the ethylene pathway are poorly understood. In the current study, we found that BRs were actively synthesized during tomato fruit ripening. We then generated transgenic lines overexpressing or silencing *SlCYP90B3*, which encodes a cytochrome P450 monooxygenase that catalyzes the rate-limiting step of BR synthesis. The expression level of *SlCYP90B3* was positively related to the contents of bioactive BRs as well as the ripening process in tomato fruit, including enhanced softening and increased soluble sugar and flavor volatile contents. Both carotenoid accumulation and ethylene production were strongly correlated with the expression level of *SlCYP90B3*, corroborated by the altered expression of carotenoid biosynthetic genes as well as ethylene pathway genes in transgenic tomato fruits. However, the application of the ethylene perception inhibitor 1-methycyclopropene (1-MCP) abolished the promotion effect of *SlCYP90B3* overexpression on carotenoid accumulation. Taken together, these results increase our understanding of the involvement of *SlCYP90B3* in bioactive BR biosynthesis as well as fruit ripening in tomato, thus making *SlCYP90B3* a target gene for improvement of visual, nutritional and flavor qualities of tomato fruits with no yield penalty.

## Introduction

Tomato (*Solanum lycopersicum*), an important agricultural crop species, is also a model system for studying fruit development and ripening. Fruit ripening in tomato is coordinated with brightened appearance caused by pigment accumulation; texture changes caused by tissue softening; and nutrient and flavor improvement through the bioactive compound, sugar, acid, and volatile organic compound (VOC) metabolism. The predominant pigments in mature tomato fruits are carotenoids, which serve as antioxidants in defense against chronic diseases and can protect against some cancers^1^. Sucrose, fructose, and glucose are the most common sugars in ripening fruits^2^. Volatiles are derived mainly from fatty acids, aliphatic amino acids, phenolic compounds, and carotenoids, including hydrocarbons, alcohols, aldehydes, esters, ethers, ketones, phenols, and sulfur- and nitrogen-containing compounds^3,4^. Among them, carotenoid-derived volatiles is usually found to exert positive effects on tomato flavor and consumer preference^5^. Carotenoid cleavage dioxygenase 1 (CCD1) contributes to the formation of volatiles, including β-ionone, geranylacetone, 6-methyl-5-hepten-2-one (MHO), and pseudoionone, in tomato fruits^4,6^. Long-term tomato breeding manipulation, such as domestication, improvement, divergence, and introgression^7^, tends to weaken alleles involved in aromatic volatile biosynthesis and strengthen alleles related to yield, disease resistance, and firmness^8^. Consequently, a reduction in the flavor of commercially produced tomato fruits has become a common consumer dissatisfaction. Many researchers have attached greater importance to the necessity and potential of improving fruit organoleptic and nutritional qualities in recent years^9^. Physiological changes in these complex biochemical characteristics during fruit ripening and quality formation occur in a highly synchronized manner and are regulated by the interaction of various factors, including external environmental stimuli and endogenous phytohormones^10^.

The onset of climacteric fruit ripening is accompanied by a burst of ethylene production that occurs in an autocatalytic manner^11^. Accordingly, ethylene acts as a master regulator that initiates and determines normal ripening of climacteric fruit^12–14^. Techniques manipulating ethylene biosynthesis, perception, or signal transduction are considered to be important strategies for modulation of fruit quality. In addition to ethylene, phytohormones such as auxin, gibberellin, abscisic acid, jasmonic acid, and brassinosteroids (BRs) have also been demonstrated to regulate carotenoid accumulation during tomato fruit ripening^10^. However, the molecular mechanisms of these phytohormones as well as their crosstalk in fruit ripening and quality formation have been less studied than those of ethylene.

**Fig. 1:**
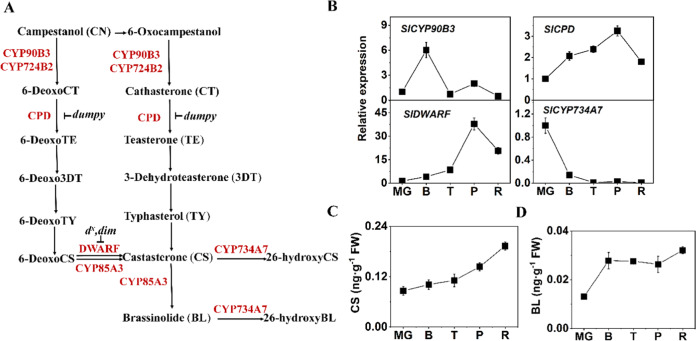
Expression levels of BR biosynthetic and catabolic genes and changes in BR contents during tomato fruit ripening. **a** BR biosynthetic and catabolic pathways in tomato. **b** Expression levels of BR biosynthetic genes (*SlCYP90B3*, *SlCPD*, *SlDWARF*) and the BR catabolic gene (*SlCYP734A7*) during tomato fruit ripening. **c** Endogenous CS content during tomato fruit ripening. **d** Endogenous BL content during tomato fruit ripening. FW, fresh weight. Three biological replicates (*n* = 3) were used for each analysis. The error bars indicate the standard deviations. MG mature green, B breaker, T turning, P pink, R red.

BRs, which comprise various kinds of polyhydroxylated steroidal phytohormones, play broad roles in plant growth and development, including stomatal development, root growth, stem elongation, leaf epinasty, vascular differentiation, and floral development as well as in plant responses to abiotic and biotic stresses^15^. Extensive studies in *Arabidopsis* with molecular genetic approaches have revealed BR biosynthetic pathways and signaling pathways from receptor kinases in the plasma membrane to transcription factors in the nucleus. However, little attention has been paid to the regulatory role of BRs in tomato fruit ripening. BR treatment of tomato fruit pericarp discs enhances lycopene content as well as ethylene production^16^. Our previous study showed that ectopic overexpression (OE) of transcription factor genes (*brassinazole resistant 1-1D*, *Atbzr1-1D*), essential components of BR signaling, enhanced carotenoid accumulation in tomato fruits^17^. To date, more than 70 natural BRs have been identified in plants^18^, among which castasterone (CS) and brassinolide (BL) are frequently identified as being widely distributed and are considered to be the most bioactive BRs in plants^19^. However, BL has not been detected in tomato seedlings, demonstrating the presence of unique BR metabolic pathways as well as a potential role of BL in tomato at the reproductive stage^20^. Unlike other phytohormones, BRs function locally and display activity at very low concentrations (nanomolar to the picomolar range), and the physiological and biochemical effects of exogenous BR application are often concentration dependent. The spatiotemporal biosynthesis of BRs is usually strictly altered to maintain proper plant growth and development. To verify the mechanism by which BRs regulate fruit ripening, we need to pay more attention to the endogenous BR biosynthetic pathway in tomato fruits. There are two common parallel biosynthetic routes for CS and BL production: the early and late C-6 oxidation pathways^21,22^. In the late C-6 oxidation pathway, campestanol (CN) is initially oxidized to 6-deoxocathasterone (6-deoxoCT), which then is successively oxidized to generate 6-deoxoteasterone (6-deoxoTE), 6-deoxo-3-dehydroteasterone (6-Deoxo3DT), 6-deoxotyphasterol (6-deoxoTY), 6-deoxocastasterone (6-deoxoCS), and CS (Fig. 1a). CS is then catalyzed to BL in the last step. Most enzymes involved in the BR biosynthetic pathway are members of the cytochrome P450 (Cyp450) superfamily (CYP85A, CYP90A, CYP90B, CYP90C, CYP90D, and CYP724B), with the exception of the reductase de-etiolated 2 (DET2)^23^. SlDWARF (SlCYP85A1), which catalyzes 6-the conversion of deoxoCS to CS, is the first identified Cyp450 within the tomato BR biosynthetic pathway, with the related deficient mutant *d*^*x*^ containing no detectable CS or BL^24^. Studies of *SlDWARF* OE have demonstrated an important role of BRs in tomato vegetative growth^20^. However, limited information is available about the function of BR biosynthetic pathway genes in fruit ripening and quality improvement.

DWF4 (CYP90B/CYP724B) catalyzes the C22 α-hydroxylation reaction in the early stage and acts as a rate-limiting enzyme in BR biosynthesis^25^. OE of *DWF4* leads to increased vegetative growth in *Arabidopsis*^26^ and *Brassica napus*^27^. Moreover, the regulation of *DWF4* expression affects plant architecture^28^, seed size, and weight^29^ in rice. The present study aims to elucidate the function of *DWF4* in tomato fruit ripening and quality formation. Fruit-specific *SlCYP90B3* (*Solyc02g085360*) was identified in tomato as an ortholog of *Arabidopsis DWF4* (Supplementary Fig. 1). *SlCYP90B3-OE* and *SlCYP90B3-RNAi* transgenic lines were generated, and a subset of fruit ripening traits was characterized. The fruit ripening process, carotenoid accumulation, and ethylene production were found to be modulated by the *SlCYP90B3* expression level. Moreover, favorable flavor volatiles was also improved in the fruits of *SlCYP90B3-OE* lines with no penalty yield. These results help to reveal the involvement of BRs in the regulation of tomato fruit ripening and the potential for manipulating *SlCYP90B3* to obtain more attractive and healthier fruits for human consumption.

## Results

### Expression of BR biosynthetic and catabolic genes and the accumulation of BRs during tomato fruit ripening

We examined the expression levels of BR biosynthetic genes (*SlCYP90B3*, *SlCPD*, *SlDWARF*) and catabolic genes (*SlCYP734A7*) during tomato fruit ripening (Fig. 1a). The expression level of *SlCYP90B3* increased rapidly and peaked at the breaker stage (B), whereas the transcripts of *SlCPD* and *SlDWARF* reached a peak at the pink stage (P) and then declined. In contrast, the expression of *SlCYP734A7* decreased throughout fruit ripening (Fig. 1b). Next, we measured endogenous bioactive BR levels: CS and BL increased gradually from the mature green (MG) stage to the red stage (R) (Fig. 1c, d). These results imply that BRs are actively synthesized during tomato fruit ripening and may play a regulatory role in fruit ripening.

### Phenotypes of *SlCYP90B3-OE* and *SlCYP90B3-RNAi* transgenic tomato fruits

**Fig. 2:**
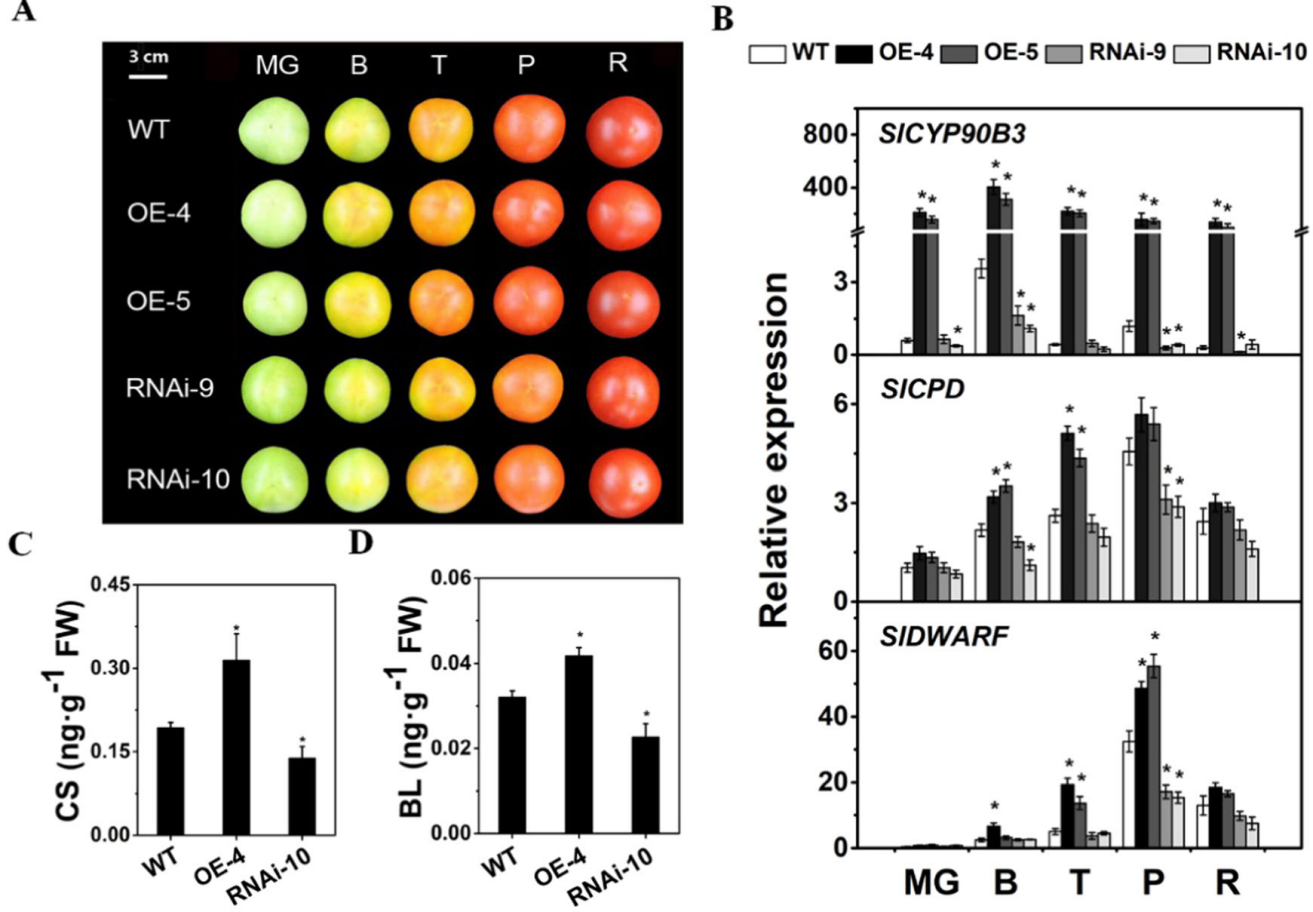
Generation of *SlCYP90B3-OE* and *SlCYP90B3-RNAi* transgenic plants. **a** Fruits at five ripening stages from mature green to red. **b** Expression levels of BR biosynthetic genes during tomato fruit ripening. **c** CS content of fruits at the R stage. **d** BL content of fruits at the R stage. The scale bar represents 3 cm. Three biological replicates (*n* = 3) were used for each analysis (*P < 0.05; Student’s *t*-test). The error bars indicate the standard deviations. WT wild type, MG mature green, B breaker, T turning, P pink, R red.

Given that SlCYP90B3 catalyzes the rate-limiting step in BR biosynthesis and that its transcript peaked at the breaker stage during fruit ripening (Fig. 1a, b), *SlCYP90B3-OE* and *SlCYP90B3-RNAi* constructs were introduced into tomato cultivar Ailsa Craig (AC). Two independent single-insert lines of both *SlCYP90B3-OE* and *SlCYP90B3-RNAi* were characterized after greenhouse cultivation for three successive generations. We noticed obvious color differences of the fruits at the T and P stages between the *SlCYP90B3* transgenic lines and the wild type. Notably, the *SlCYP90B3-OE* fruits at the T and P stages showed a stronger pigmentation phenotype according to visual observations (Fig. 2a). The expression levels of *SlCYP90B3*, *SlCPD* and *SlDWARF* increased in *SlCYP90B3-OE* transgenic lines and decreased in the *SlCYP90B3-RNAi* transgenic lines (Fig. 2b). To further verify whether endogenous levels of BRs were altered in the *SlCYP90B3* transgenic plants, the BR contents were measured. The results showed that the levels of both CS and BL were significantly elevated in the *SlCYP90B3-OE* transgenic lines but markedly decreased in the *SlCYP90B3-RNAi* transgenic lines (Fig. 2c, d).

**Fig. 3:**
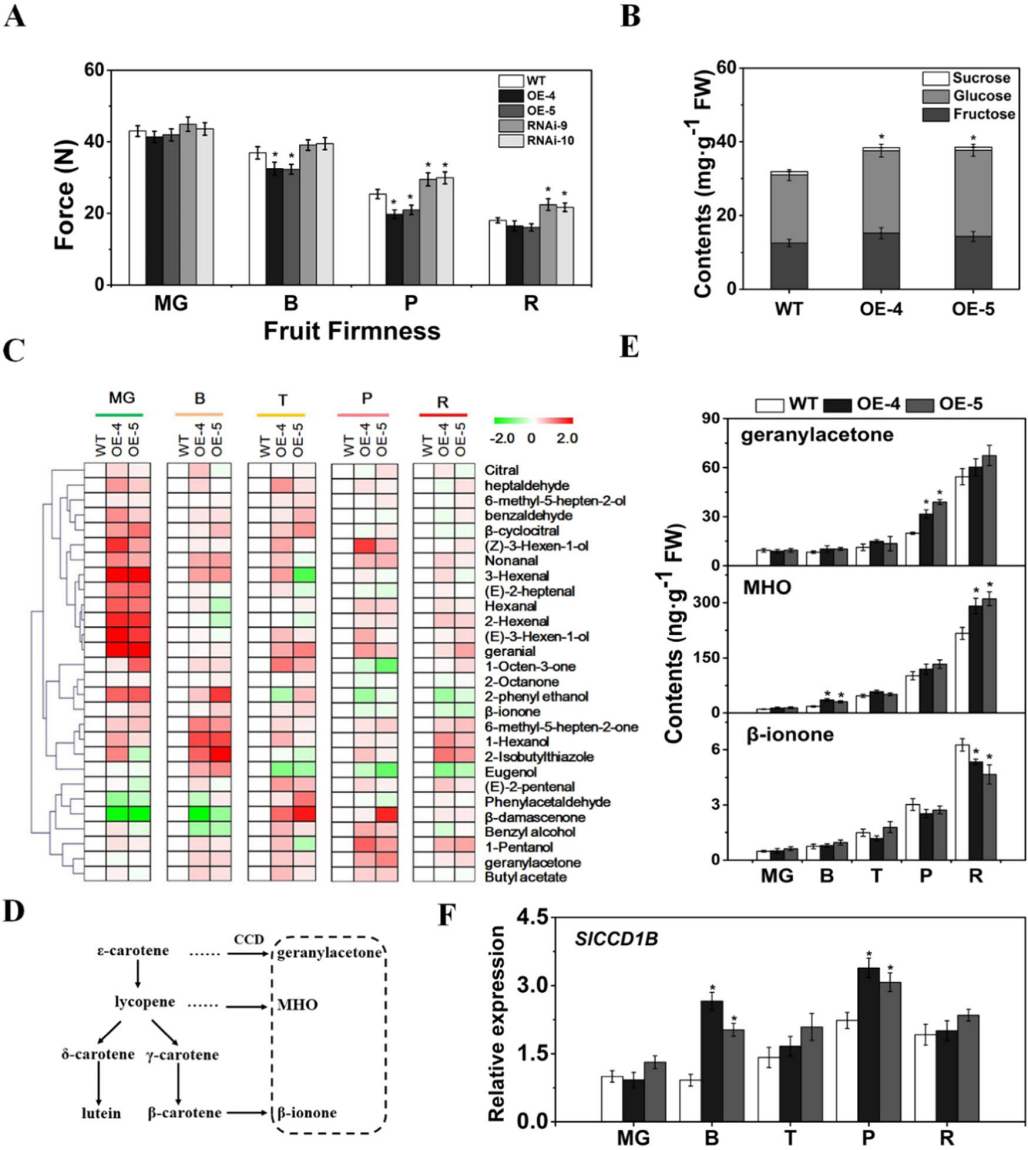
Overexpression of *SlCYP90B3* promotes tomato fruit ripening. **a** Fruit firmness at the MG, B, P and R stages. **b** Contents of soluble sugars in wild-type and *SlCYP90B3-OE* fruits at the R stage. **c** Heatmap visualization of volatiles in wild-type and *SlCYP90B3-OE* fruits during tomato fruit ripening. **d** Volatiles derived from carotenoids. **e** Contents of geranyl acetone, MHO and β-ionone during tomato fruit ripening. **f** The expression level of *SlCCD1B*. Three biological replicates (*n* = 3) were used for each analysis (*P < 0.05; Student’s *t*-test). The error bars indicate the standard deviations. WT wild type, MG mature green, B breaker, T turning, P pink, R red.

Both *SlCYP90B3-OE* transgenic lines showed decreased fruit firmness at the B and P stages, while both *SlCYP90B3-RNAi* transgenic lines showed increased fruit firmness at the P and R stages (Fig. 3a). Compared with those in the wild-type fruits, the contents of total sugars in the *SlCYP90B3-OE* transgenic fruits were significantly higher at the R stage (Fig. 3b).

Volatile composition and content are desirable traits in tomato fruits and promote consumer palatability^2,30^. Twenty-eight-volatile compounds were detected in tomato fruits during ripening: 12 lipid-derived volatiles, 8 carotenoid-derived volatiles, 2 phenylalanine-derived volatiles, 3 phenylpropanoid-derived volatiles and 3 branched chain-derived volatiles (Fig. 3c, Supplementary Table 1). The total contents of the volatiles increased during fruit ripening, and compared with the wild type, the *SlCYP90B3-OE* lines accumulated more volatiles at the MG, P, and R stages (Supplementary Fig. 2).

According to previous reports, carotenoid-derived volatiles are essential to tomato flavor and promote consumer palatability^5,8^, and their biosynthesis from carotenoids has been well studied (Fig. 3d). Geranylacetone and MHO, which are catalyzed by oxidative cleavage of lycopene, are carotenoid-derived volatiles abundantly present in tomato fruits. The fruits of *SlCYP90B3-OE* lines at the B and R stages showed increased levels of MHO (Fig. 3e). Moreover, the content of geranylacetone also increased at the P stage in the *SlCYP90B3-OE* lines compared with the wild type (Fig. 3e). However, the β-ionone level decreased at the R stage in the *SlCYP90B3-OE* fruits (Fig. 3e). CCD1B cleaves carotenoids to produce carotenoid-derived volatiles, which markedly increased at the B and P stages in the *SlCYP90B3-OE* fruits (Fig. 3f). Taken together, these results suggest that increased carotenoid-derived volatiles caused by *SlCYP90B3* overexpression may be due to enhanced expression of *SlCCD1B*.

### ***SlCYP90B3*** OE promotes carotenoid accumulation in fruits

**Fig. 4:**
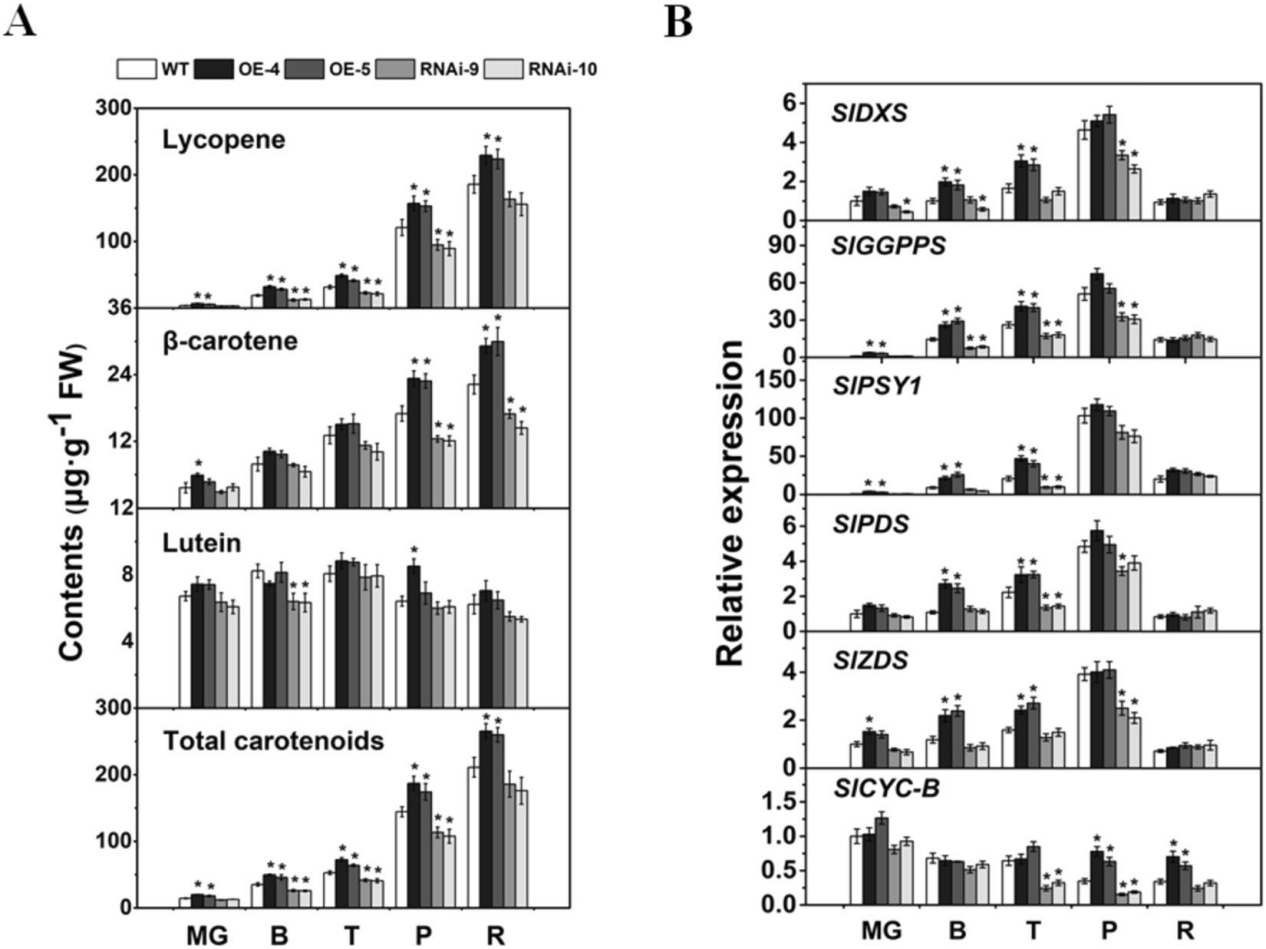
Carotenoid contents and expression levels of carotenoid biosynthetic genes in wild-type, *SlCYP90B3-OE*, and *SlCYP90-RNAi* transgenic fruits. **a** Carotenoid contents in tomato fruits. **b** Expression levels of carotenoid biosynthetic genes during tomato fruit ripening. Three biological replicates (n = 3) were used for each analysis (*P < 0.05; Student’s *t*-test). The error bars indicate the standard deviations. WT wild type, MG mature green, B breaker, T turning, P pink, R red.

To determine the causes of the color changes of the fruits of the transgenic lines, the carotenoid (lycopene, β-carotene, and lutein) contents in the fruits of the *SlCYP90B3* transgenic lines and the wild type at five typical developmental stages were analyzed. The level of lycopene significantly increased in both *SlCYP90B3-OE* transgenic lines at all five stages, and the β-carotene level significantly increased at the P and R stages. In contrast, a significant decrease in lycopene content at the B, T, and P stages, along with a significant decrease in β-carotene content at the P and R stages, was observed in the two *SlCYP90B3-RNAi* transgenic lines. A significant change in lutein content was observed only between the *SlCYP90B3*-*RNAi* transgenic lines and the wild type at the B stage (Fig. 4a). The carotenoid biosynthetic pathway in tomato has been well elucidated. 1-Deoxy-D-xylulose-5-phosphate synthase (DXS), geranylgeranyl pyrophosphate synthase (GGPPS), phytoene synthase 1 (PSY1), phytoene desaturase (PDS), β-carotene desaturase (ZDS) and β-cyclase (CYC-B) are the committed enzymes involved in carotenoid biosynthesis in tomato fruits. All five lycopene biosynthetic genes (*SlDXS*, *SlGGPPS*, *SlPSY1*, *SlPDS*, and *SlZDS*) presented the same expression patterns; their expression significantly increased in the *SlCYP90B3-OE* transgenic lines and decreased in the *SlCYP90B3*-*RNAi* transgenic lines (Fig. 4b). Among them, the expression levels of *SlDXS*, *SlPDS*, and *SlZDS* were elevated at the B and T stages in the *SlCYP90B3-OE* lines, and those of two other genes (*SlGGPPS*, *SlPSY1*) were significantly elevated at the MG, B, and T stages (Fig. 4b). The transcript level of *SlGGPPS* decreased at three sequential stages (B, T, and P) in the *SlCYP90B3-RNAi* transgenic lines compared to the WT. The mRNA level of *SlCYC-B* increased at the P and R stages in the fruits of the *SlCYP90B3-OE* transgenic lines. Moreover, the expression level of *SlCYC-B* significantly decreased at the T and P stages in the fruits of the *SlCYP90B3-RNAi* transgenic lines (Fig. 4b). These results suggest that OE of *SlCYP90B3* is related to increased carotenoid accumulation in tomato fruits.

### OE of *SlCYP90B3* enhances ethylene production

**Fig. 5:**
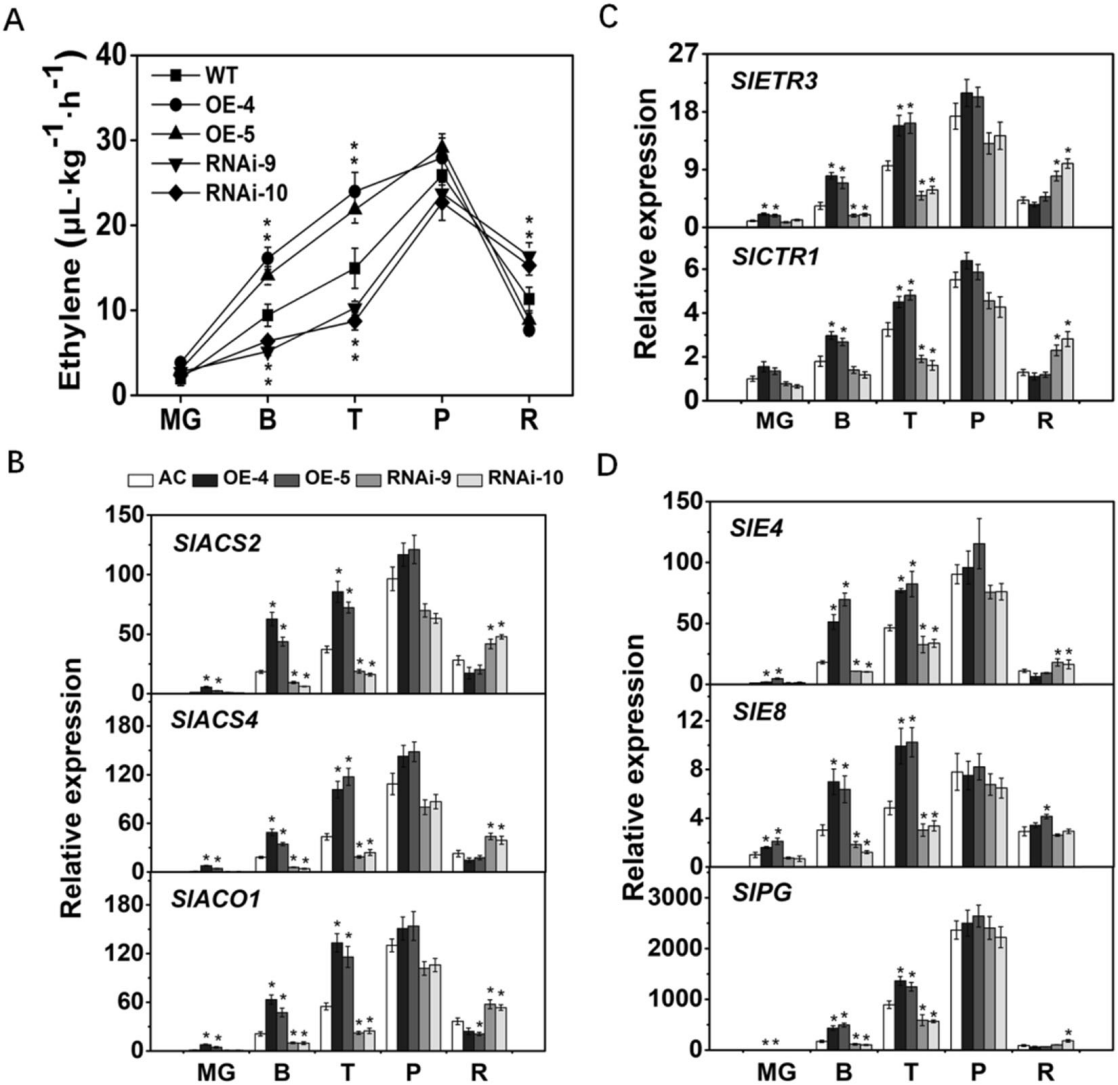
Ethylene production and responses in *SlCYP90B3-OE* and *SlCYP90-RNAi* transgenic fruits. **a** Ethylene production during tomato fruit ripening. **b** Expression levels of ethylene biosynthetic genes during tomato fruit ripening. ACS2, ACC synthase 2; ACS4, ACC synthase 4; ACO1, ACC oxidase 1; **c** Expression levels of genes involved in ethylene signaling during tomato fruit ripening. ETR3, ethylene receptor 3; CTR1, constitutive triple response 1. **d** Expression levels of ethylene response genes during tomato fruit ripening. E4, peptide Met sulfoxide reductase; E8, 1-aminocyclopropane-1-carboxylate oxidase-like protein; PG, polygalacturonase. Three biological replicates (*n* = 3) were used for each analysis (*P < 0.05; Student’s *t*-test). The error bars indicate the standard deviations. WT wild type, MG mature green, B breaker, T turning, P pink, R red.

Ethylene is usually considered the master regulator of fruit ripening; thus, ethylene production was measured to verify the effects of SlCYP90B3 on tomato fruit ripening. It was found that ethylene production increased at the B and T stages and peaked at the P stage in both the *SlCYP90B3* transgenic lines and the wild type. Notably, higher ethylene content was observed in the *SlCYP90B3-OE* fruits at the B and T stages than in the wild-type fruits (Fig. 5a). These findings were consistent with elevated carotenoid levels and decreased firmness in the OE lines (Figs. 3a, 4a). We also examined the expression levels of ethylene biosynthesis and signaling genes and found that the expression of the ethylene biosynthetic genes (*SlACS2*, *SlACS4*, and *SlACO1*) significantly increased in the *SlCYP90B3-OE* transgenic lines at the MG, B and T stages (Fig. 5b). The expression levels of genes involved in ethylene signaling, including *SlETR3* (encoding ethylene receptor) and *SlCTR1* (encoding Raf-like kinase), also significantly increased (Fig. 5c). Similarly, ethylene response genes (*SlE4*, *SlE8*, and *SlPG*) were also upregulated in the *SlCYP90B3-OE* transgenic lines at the MG, B and T stages (Fig. 5d). Taken together, these results suggest that OE of *SlCYP90B3* enhanced both ethylene biosynthesis and signaling in tomato fruits.

### SlCYP90B3 promotes carotenoid accumulation in an ethylene-dependent manner

**Fig. 6:**
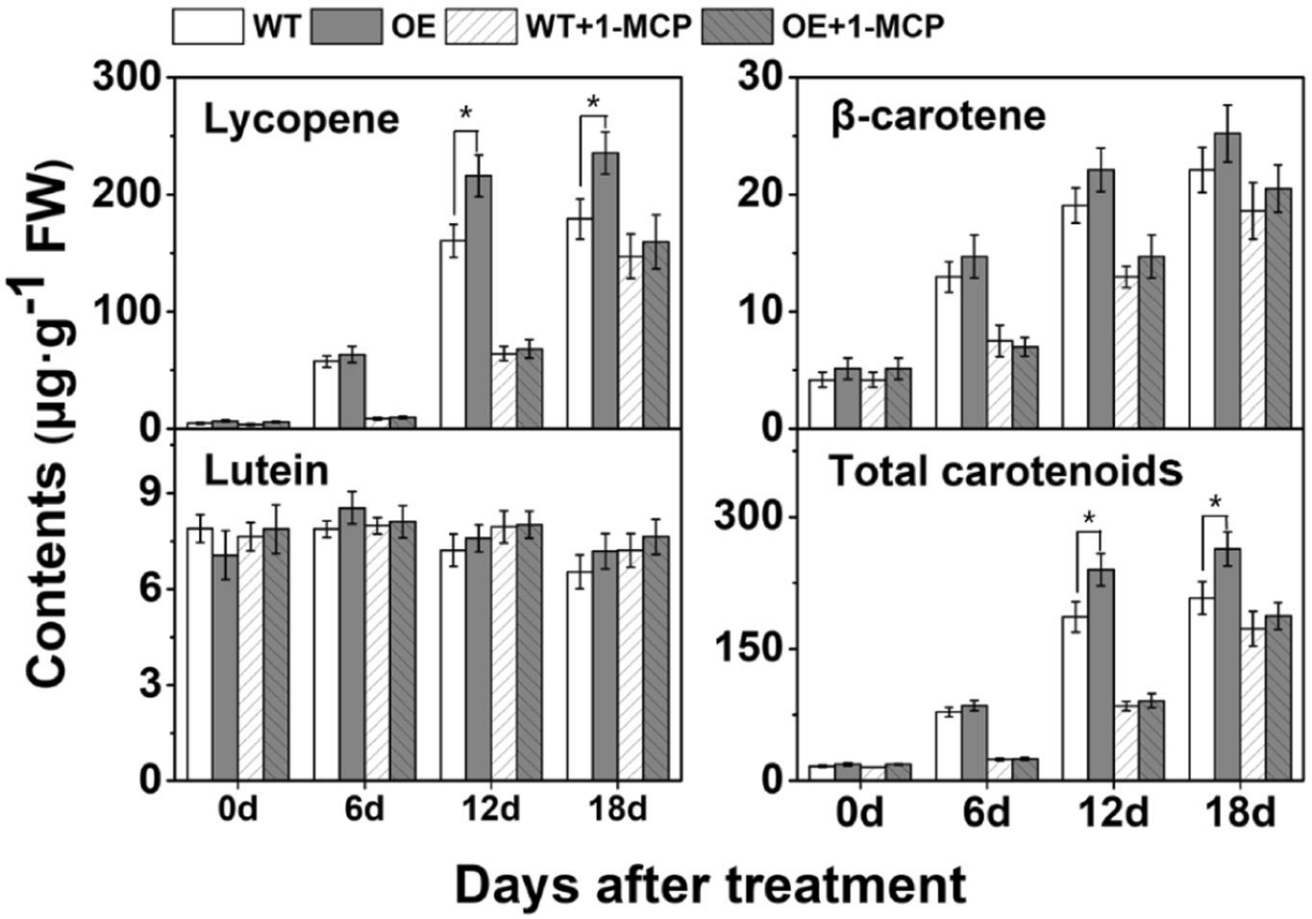
Carotenoid accumulation in wild-type and *SlCYP90B3-OE* fruits with or without 1-MCP treatment. Here, 0.5 μL L^−1^ 1-MCP was applied as treatment. Three biological replicates (*n* = 3) were used for each analysis (*P < 0.05; Student’s *t*-test). The error bars indicate the standard deviations.

Here, we used 1-MCP, an effective ethylene perception inhibitor, to further explore the interaction between BRs and ethylene in promoting carotenoid accumulation. The contents of lycopene and total carotenoids increased in the *SlCYP90B3-OE* fruits (OE-4) after 12 d and 18 d of storage compared with those in the wild-type fruits (Fig. 6), while no significant increase in lutein or β-carotene accumulation in the *SlCYP90B3-OE* (OE-4) fruits were observed after postharvest storage. The accumulation of lycopene and β-carotene was significantly repressed by 1-MCP treatment at 6 d and 12 d of storage in both *SlCYP90B3-OE* and wild-type fruits, and the promotion of lycopene and total carotenoid accumulation in the *SlCYP90B3-OE* (OE-4) fruits were inhibited by 1-MCP treatment (Fig. 6). These results imply that *SlCYP90B3* OE promotes carotenoid accumulation during tomato fruit ripening in an ethylene-dependent manner.

### OE of *SlCYP90B3* improves tomato fruit yield

After fruit quality evaluation, we measured the fruit yield of *SlCYP90B3-OE* and *SlCYP90B3-RNAi* lines grown in the greenhouse and found that fruit development after pollination was accelerated in the *SlCYP90B3-OE* lines and delayed in the *SlCYP90B3-RNAi* lines in comparison with that in the wild type (Supplementary Fig. 3, Table 1). We then quantified the number of total fruits per plant at 135 days after sowing and found that the *SlCYP90B3-OE* plants produced more fruits than did the wild-type and *SlCYP90B3-RNAi* transgenic plants, but no significant difference in weight per fruit was detected among any of the transgenic lines or wild type. Moreover, the space required for the *SlCYP90B3-OE* transgenic plants decreased as a consequence of a decreased canopy; thus, the fruit yield of the OE-4 and OE-5 plants increased by 39.6% and 29.9%, respectively, on an area basis. Thus, yield improvements could be achieved by the high-density planting of *SlCYP90B3-OE*. These results indicate that *SlCYP90B3* has the potential to improve both the quality and yield of tomato fruits.

**Table 1:** Fruit development and yield of wild-type and *SlCYP90B3* transgenic lines.

Parameter	WT	OE-4	OE-5	RNAi-9	RNAi-10
Days from anthesis to fruit ripening	52.4 ± 1.7 b	47.6 ± 1.2 c	46.8 ± 1.3 c	57.1 ± 1.5 a	58.3 ± 1.9 a
Fruit number per plant	18.4 ± 1.6 b	22.2 ± 1.3 a	21.9 ± 1.3 a	14.7 ± 1.5 c	15.1 ± 1.2 c
Weight per fruit (g)	29.7 ± 5.8 a	25.9 ± 5.5 a	26.7 ± 4.4 a	34.6 ± 6.2 a	35.9 ± 5.1 a
Fruit yield per plant (g)	560.5 ± 58.2 a	521.9 ± 68.8 a	538.9 ± 78.4 a	496.6 ± 60.2 a	474.1 ± 64.5 a
Estimated fruit yield (kg m^−2^)	4.12 ± 0.44 b	5.75 ± 0.69 a	5.35 ± 0.58 a	2.91 ± 0.36 c	3.02 ± 0.47 c

The values shown are the means ± SDs of 15 plants. The means denoted by the same letter do not differ significantly according to ANOVA in conjunction with Duncan’s test (*P* < 0.05).

## Discussion

While increases in carotenoids and a sweet aroma during tomato fruit ripening are considered a wise means to promote seed dispersal, humans domesticate tomato as an important diet source with a focus on uniformity, size, yield, and shelf-life^31^. Currently, improvements in traditional organoleptic and functional characteristics in addition to external appearance are being reconsidered by breeders because consumers are paying increased amounts of attention to the flavor and health benefits of foods. A complete picture and functional annotation of the tomato genome, as well as genetic engineering, will not only offer improved potential but also accelerate the development of new crop varities^32,33^.

**Fig. 7:**
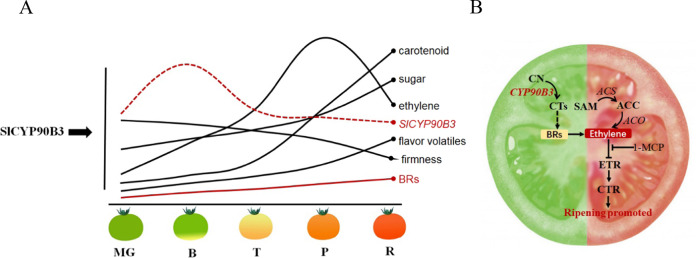
A proposed model of the involvement of the BR biosynthetic gene SlCYP90B3 in tomato fruit ripening in an ethylene-dependent manner. **a** During tomato fruit ripening, morphological and biochemical changes take place, including gradually elevated BR levels, sugar and carotenoid accumulation, enhanced release of ethylene and flavor volatiles, and reduced firmness. *SlCYP90B3* overexpression promotes all aspects of the ripening process. MG, mature green; B, breaker; T, turning; P, pink; R, red. **b** SlCYP90B3 catalyzes the rate-limiting step from campestanol to cathasterone/6-deoxocathasterone in the BR biosynthetic pathway and increases the level of active BRs. The induced BRs in turn regulate the biosynthesis of ethylene and its downstream signaling to promote fruit ripening. CN campestanol, CTs cathasterone and 6-deoxocathasterone, BRs brassinosteroids, SAM S-adenosyl methionine, ACC 1-aminocyclopropane-1-carboxylic acid, ACS ACC synthase, ACO ACC oxidase.

In this study, the contents of bioactive BRs, CS, and BL, were found to gradually increase during tomato fruit ripening (Fig. 1c, d). Similarly, the presence of endogenous BRs at relatively high levels has been also been observed during the strawberry fruit-coloring period^34^ and at the onset of grape (*Vitis vinifera*) ripening^35^. The apparent BR accumulation in the fruits raises the possibility of a specific role of BRs in fruit ripening. To gain more insight into the regulatory mechanisms underlying fruit ripening, we manipulated the expression of *SlCYP90B3* and analyzed its global effects on tomato ripening, including firmness, sugar content, flavor volatile contents, carotenoid accumulation, ethylene production, and yield. *SlCYP90B3* OE in tomato fruit played a broad regulatory role in tomato improvement, which also included affecting fruit quality and yield. Manipulation of *SlCYP90B3* with altered levels of bioactive BR contents and ethylene production in the current study helps to understand how BR grid-like biosynthetic routes are finely regulated and to understand the coordination with ethylene during tomato fruit ripening (Fig. 7).

### Fine tuning of BR biosynthesis during tomato fruit ripening

Metabolic pathways in plants are interconnected and display interdependencies to enhance or suppress each other. BRs generally stimulate growth and development at low concentrations without active transportation, and plants are sensitive to excessive amounts of BRs^15^. Consequently, plants have evolved strategies to maintain BR homeostasis and normal function. The first strategy involves distinct developmental regulation. During vegetative growth, CS, but not BL, was produced by SlCYP85A1 (SlDWARF), and CS exerts biological activity^24,36–38^. SlCYP85A3, which, interestingly, is expressed in fruits, catalyzes the same reaction as SlCYP85A1 for CS production, while BL biosynthesis from CS was also detected^39^. SlCYP724B2 and SlCYP90B3 are functionally redundant in the early steps of BR biosynthesis in tomato. In immature fruits, only *SlCYP90B3* mRNA was detected, and the expression level of the *SlCYP90B3* transcript was higher than that of the *SlCYP724B2* transcript in young leaves and mature fruits^25^. During fruit ripening, *SlCYP90B3* expression was found to respond transiently at the B stage, whereas the mRNA levels of *SlCPD* and *SlDWARF* gradually increased from the B to P stages (Fig. 1b). The second strategy involves feedback regulation. Exogenous BR treatment, as well as the increase in endogenous BR levels and active BR signaling, negatively regulates BR biosynthetic genes via a feedback loop^40^. Although the contents of endogenous CS and BL in *SlCYP90B3-OE* fruits increased, the expression levels of *SlCPD* and *SlDWARF* were upregulated rather than repressed (Fig. 2b–d). It could be that SlCYP90B3 participates in the early step of BR biosynthesis and that the feedforward mechanism overcomes the feedback. The third strategy involves the deactivation or degradation of BRs. SlCYP734A7 functions in BR catabolism in tomato^41^. The expression level of *SlCYP734A7* dramatically decreased, affording increased levels of bioactive BRs (CS and BL) during fruit ripening (Fig. 1b–d). The tactics within plants could call for a well-orchestrated modulation of BR biosynthesis and catabolism.

### BR and ethylene work together to promote carotenoid accumulation during fruit ripening

OE of *SlCYP90B3* was found to promote the accumulation of lycopene and β-carotene, as well as total carotenoids, via an orderly transcriptional regulation of genes involved in carotenoid biosynthesis (Fig. 4). Considering the central role of ethylene in fruit ripening, we investigated the influence of altered endogenous BR levels by manipulation of *SlCYP90B3* on ethylene production (Fig. 5a), as well as the expression of genes involved in ethylene biosynthesis, ethylene signaling, and the ethylene response (Fig. 5b–d). The results revealed that *SlCYP90B3* OE enhanced both ethylene biosynthesis and signaling, which contributes to BR-enhanced carotenoid accumulation in tomato fruits. To further verify whether BRs regulate carotenoid accumulation in an ethylene-dependent manner, we treated *SlCYP90B3-OE* transgenic fruits with 1-MCP, the most effective inhibitor of ethylene action, to rule out the involvement of ethylene. Surprisingly, the enhancement of carotenoid accumulation by *SlCYP90B3* OE was eliminated by 1-MCP treatment (Fig. 6), suggesting that BR-promoted carotenoid accumulation is ethylene dependent. In our previous work, we applied 3.0-μM EBL (2,4-epibrassinolide) to pericarp discs of a tomato ethylene receptor mutant, *Never ripe* (*Nr*) in the Pearson (PSN) background, and still observed an increase in the contents of lycopene and β-carotene in *Nr*; the increase seemed to be ethylene independent^17^. The contrasting results between the application of exogenous BRs to the ethylene receptor mutant and the application of 1-MCP to the *SlCYP90B3-OE* transgenic line are presumably due to the different conditions used. Additionally, the possibility that *Nr* plants retain residual ethylene responsiveness because of the partia1 dominance of the receptor gene (*SlETR3*) cannot be ruled out^42^. Interestingly, a large number of BR and ethylene coregulated genes have been detected in *Arabidopsis*, and these genes are enriched in BR-regulated BZR1 (an essential component of BR signaling) targets^43^. Moreover, BZR1 directly regulates several genes involved in ethylene biosynthesis and signaling^43^. A model by which the ethylene transcription factor EIN3 activates the MADS-box transcription factor RIN-TAGL1 to form a positive feedback loop amplifying autocatalytic ethylene synthesis has been illustrated^44^. The ripening process operates after the fruit reaches its final size at the MG stage, so the initial induction of ripening should be associated with both environmental and developmental cues. Both ethylene biosynthesis and signaling pathways are conserved in plants; thus, the BR-regulated BZR1 targets of ethylene biosynthetic genes should contribute to BR-promoted ethylene production in tomato fruits (Fig. 5). Moreover, *SlCYP90B3* OE also shortened the tomato fruit growth period (Table 1). These results raise the possibility that BR functions via an ethylene-dependent mechanism and that *SlCYP90B3* OE enhances ethylene-mediated ripening events in tomato fruit, including carotenoid accumulation, softening, and aromatic volatile emissions.

### Effects of *SlCYP90B3* manipulation on tomato fruit softening

Disassembly of cell wall components during ripening leads to fleshy fruit softening. The promotion of softening has been observed in exogenous EBL-treated climacteric fruits, including mango (*Mangifera indica* L.)^45^ and persimmon (*Diospyros kaki* L.)^46^. Consistent with the effects of exogenous EBL-treatment, softening was also enhanced by *SlCYP90B3* OE, with elevated BR levels, and was inhibited by *SlCYP90B3* silencing, with reduced BR levels, in the present study (Fig. 3a). However, in both the EBL-treated mango and persimmon, as well as in tomato with elevated endogenous BR levels, ethylene production was promoted^45,46^ (Fig. 5a), indicating an indirect role for BRs in climacteric fruit softening. However, the application of EBL to nonclimacteric grape^35^ and strawberry^34^ still significantly induced fruit ripening. In addition, BRs have been shown to activate several genes that encode cell wall-degrading enzymes and that are BZR1 targets, such as polygalacturonase, pectinesterase, pectate lyase, endo-1,4-β-glucanase, and β-galactosidase^43^. Overall, BR-regulated fruit softening may be achieved either by directly modulating cell wall degradation-related genes or by crosstalk with ethylene and other signaling pathways. Further research is needed to elucidate how BRs regulate fruit softening via their signaling and response components.

### *SlCYP90B3* OE improves flavor via modulation of volatile production

The profile of each fruit flavor is unique, which determines its specific flavor. The limited knowledge of biosynthetic pathways and regulatory networks of flavor-related volatiles is a challenge with respect to maintaining and improving flavor. Tomato volatiles are derived mainly from fatty acids, carotenoids and amino acids. Via applications of large consumer panels and analytical chemistry, a previous study identified 28 volatiles correlated with consumer liking^8^. The levels of 2-hexenal, 1-pentanol, and 1-hexanol from lipids; MHO and geranial from carotenoids; and 2-isobutylthiazole from branched-chain amino acids were elevated in *SlCYP90B3-OE* tomato fruits (Supplementary Table 1). Among them, 1-pentanol, MHO and 2-isobutylthiazole contribute positively to consumer preferences^2^. The volatiles are active at nanomolar to picomolar concentrations, and even a slight increase in their content can lead to significant flavor enhancement. The tomato pangenome construction and analyses with phylogenetically and geographically representative accessions reveal that the positive regulation of apocarotenoid production contributes to desirable tomato flavor^33^. Apocarotenoid volatiles derived from carotenoids through SlCCD are typical in ripening tomato fruits (Fig. 3d). *SlCCD1B*, which accumulates throughout the ripening process^6^, encodes an enzyme that is more active than *SlCCD1A* is. In agreement with the elevated contents of geranylacetone and MHO, the expression level of *SlCCD1B* increased in response to *SlCYP90B3* OE (Fig. 3f). However, a reduction in β-ionone was observed in *SlCYP90B3-OE* tomato fruits at the R stage (Fig. 3e). This may have occurred because β-ionone is the substrate of other chemicals. Another possibility is that SlCCD1B has different affinities for different substrates, and the metabolic flux to β-ionone accumulation could, therefore, be weakened. The enhanced transcription of *SlCCD1B* might be attributed to increased BR levels or by the feedforward accumulation of carotenoids in *SlCYP90B3-OE* fruits.

## Experimental procedures

### Plant materials and growth conditions

Tomato (*Solanum lycopersicum* cv. Ailsa Craig) plants, including wild-type and two *SlCYP90B3-OE* (OE-4, OE-5) lines and *SlCYP90B3-RNAi* (RNAi-9, RNAi-10) lines, were grown in a greenhouse at 25/18 °C (day/night). The ripening stages (MG, B, T, P, R) of fruits were defined based on fruit color, as described previously^47^. There were three replicates per treatment, with six fruits per replicate. Fresh fruits were harvested to analyze the ethylene contents and firmness and then frozen in liquid nitrogen for subsequent RNA extraction and metabolite profiling.

### Transgenic plant construction

*SlCYP90B3-OE* and *RNAi* constructs were generated using pGWB17 and pBIN19 vectors, respectively, as described previously^48^. For the construction of the OE construct, the full-length coding sequence of *SlCYP90B3* was inserted downstream of the 35 S promoter in pGWB17 by Gateway cloning (Invitrogen, USA). For *RNAi* vector construction, a 477 bp fragment derived from the cDNA of *SlCYP90B3* was synthesized via PCR and then fused into the plasmid pHANNIBAL in both antisense and sense orientations. The *SlCYP90B3* RNA interference fragment was recombined to the plant binary vector pBIN19, generating *SlCYP90B3-RNAi* (Supplementary Table 2). The OE and RNAi vectors were then introduced into *Agrobacterium tumefaciens* strain LBA4404 (Tiangen, Beijing, China), and tomato transformation was performed as described^49^. Both independent OE (OE-4, OE-5) and RNAi (RNAi-9, RNAi-10) single-insertion lines were subsequently assessed after cultivation for three successive generations.

### RNA extraction and qRT-PCR-based analysis

Total RNA was isolated from tomato fruits by the use of a TRIzol Reagent Kit (Takara Bio, Otsu, Japan), and 2% (w/v) agarose gel was used to evaluate RNA integrity. One microgram of RNA was reverse-transcribed into cDNA using a PrimeScript cDNA Synthesis Kit (Takara, Japan). qRT-PCR was then performed using an iCycler (Bio-Rad, USA), and the gene-specific primers used are shown in Supplementary Table 3. The relative gene expression was calculated according to the 2^–ΔΔCT^ method^50^.

### HPLC-MS/MS analysis of BRs

The levels of bioactive endogenous BRs (CS and BL) were measured according to methods in a previous report^51^. Approximately 2.0 g of tomato fruit flesh was ground into a powder in a precooled centrifuge tube and then extracted with 80% (v/v) methanol. The mixture was shaken at 4 °C for 2 h and then centrifuged at 12,000 rpm for 10 min at 4 °C. The supernatant was then eluted through prepacked Bond Elut (Agilent, USA), eluted with 3 mL of methanol, eluted through a Strata-X membrane filter (Phenomenex, USA), eluted with 3 mL of methanol again and then evaporated to dryness under a nitrogen-blowing instrument. Afterward, 200 μL of methanol was added, and the solution was filtered through a 0.22 μm membrane. Five microliters were subsequently subjected to HPLC-tandem mass spectrometry (HPLC-MS/MS) analysis. The contents of BRs were determined according to the concentration curve of BR standards (CS, BL; Sigma, USA).

### 1-MCP treatment

Sixty fruits of wild-type and *SlCYP90B3-OE* (OE-4) plants were harvested at the MG stage. For 1-MCP treatment, the fruits were placed in 25 L containers and treated with 0.5 μL L^−1^ 1-MCP for 16 h. After 1-MCP treatment, the fruits were removed from the containers and stored at 25 ± 1 °C.

### Measurement of ethylene production

Ethylene production was detected according to the methods of a previous report^52^. Five fruits were enclosed in a 2.0 L sealed container and then incubated at 20 °C for 1.5 h, after which 1 mL of the headspace gas was collected and immediately injected into a gas chromatograph (Shimadzu GC-17A, Kyoto, Japan).

### HPLC analysis of carotenoids

The extraction and analysis of carotenoids were performed as previously described via high-performance liquid chromatography (Shimadzu, Kyoto, Japan), with slight modifications^52^. Approximately 0.5 g of tomato fruit powder was extracted with 30 mL of hexane:acetone:ethanol (1:1:1, v/v/v). The extracts were then centrifuged at 12,000 g for 15 min, after which 15 mL of double distilled water was added. Three milliliters of the upper layer was eluted through a 0.22 μm filter membrane and evaporated to dryness under a nitrogen-blowing instrument. The residue was then dissolved in 1.5 mL of tetrahydrofuran:acetonitrile:methanol (15:30:55, v/v/v), after which the solutions were loaded into a liquid phase bottle for HPLC analysis. The contents of the carotenoids were determined according to the concentration curve of standards (lutein, lycopene, and β-carotene; Sigma, USA).

### SPME-GC/MS analysis of volatiles

The volatile contents were analyzed according to the methods of a previous report^53^. Five grams of fruit powder was put into a 10 mL headspace bottle, and then 5 mL of saturated NaCl solution and 2-octanol were added as internal standards. The samples were subsequently fully vortexed. The volatiles were extracted with a fiber coated with 50/30 μm of divinylbenzene/carboxen/polydimethylsiloxane (DVB/CAR/PDMS) (Supelco, Bellefonte, PA, USA) for SPME-GC-MS analysis. The NIST 8.0 database (NIST/EPA/NIH, USA) was used to match the volatiles, and authentic standards were used to compare retention times. Quantification of the compounds was performed using the peak area of the internal standard as a reference based on the total ion chromatogram (TIC).

### Firmness determination

Fruits were harvested from wild-type and transgenic lines at each developmental stage. Firmness was measured at three points on each fruit using a black plastic probe (7.5 mm) and a TA-XT2i texture analyzer (Stable Micro Systems Ltd., Godalming, UK). The puncture depth was set to 10 mm, and the unit of firmness was measured in Newtons.

### Sugar content measurements

The contents of glucose, sucrose, and fructose in tomato fruits were analyzed according to the methods of a previous report^54^. Approximately 0.1 g of tomato fruit powder was extracted in 1.5 mL of methanol and shaken at 950 rpm for 15 min at 70 °C, followed by centrifugation at 11,000 rpm for 10 min. The supernatant was successively supplemented with 1.5 mL of double distilled water and 750 μL of chloroform, after which the mixture was thoroughly vortexed. The internal standard ribitol was added, and the mixture was vacuum dried at room temperature. The derivatization process was as follows: 60 μL of freshly prepared methoxyamine hydrochloride was added and shaken at 950 rpm for 1.5 h at 37 °C; afterward, 40 μL of NSTFA was added, and the solution was shaken at 950 rpm for 0.5 h at 37 °C. The solutions were ultimately loaded into liquid phase bottles for GC analysis.

### Statistical analysis

The experimental data were analyzed with SPSS 19.0 software. Pairwise comparisons were computed using Student’s *t*-test (*P* < 0.05), while multiple comparisons were subjected to ANOVA using Duncan’s test. Statistically significant differences (*P* < 0.05) are indicated by different lowercase letters.

The volatile data were normalized to the wild-type data with log_2_ transformation and visualized as a heatmap with hierarchical clustering performed by MultiExperiment Viewer 4.9 software.

## Supplementary information

Supplementary Figures S1-S3+Tables S1-S3
