# Supplementary material for: Regulation of fruit ripening by the brassinosteroid biosynthetic gene SlCYP90B3 via an ethylene-dependent pathway in tomato
Source: Hortic Res. 2020 Oct 1;7:163. doi: 10.1038/s41438-020-00383-0 (PMC7527449; doi:10.1038/s41438-020-00383-0)
Supplement: Supplementary file 1 — Supplementary Figures S1-S3+Tables S1-S3 [file 41438_2020_383_MOESM1_ESM.docx]

**Supplementary Table 1. Contents of volatiles in wild type and *SlCYP90B3-OE* transgenic fruits at red stage.**

**VOCs Content (ng g^-1^) at R stage precursors**

**WT OE-4 OE-5**

| Hexanal | 744.00 ±57.18 a | 876.54 ±63.23 a | 815.12 ±74.48 a | Lipid |
| --- | --- | --- | --- | --- |
| (E)-2-Pentenal | 28.37 ±2.45 b | 38.39 ±3.14 a | 32.51 ±3.30 ab | Lipid |
| 3-Hexenal | 28.73 ±3.32 b | 41.33 ±3.06 a | 26.03±3.03 b | Lipid |
| heptaldehyde | 36.43 ±2.61 b | 33.02 ±1.48 b | 42.98 ±2.07 a | Lipid |
| 2-Hexenal | 506.31 ±53.66 b | 684.80±4.89 a | 639.92 ±56.66 a | Lipid |
| 1-Pentanol | 9.39 ±0.91 b | 14.24 ±2.98 a | 15.41±3.22 a | Lipid |
| 1-Octen-3-one | 22.39 ±2.61 a | 23.32 ±2.21 a | 27.63±3.64 a | Lipid |
| (E)-2-heptenal | 100.90± 9.60 a | 109.85±5.37 a | 100.93±12.55 a | Lipid |
| 1-Hexanol | 21.86 ±2.03 c | 40.32 ±1.52 a | 30.69 ±1.26 b | Lipid |
| (E)-3-Hexen-1-ol | 1.71±0.19 a | 1.83 ±0.15 a | 2.08 ±0.42 a | Lipid |
| (Z)-3-Hexen-1-ol | 26.59 ±1.09 a | 27.16 ±1.94 a | 30.59 ±2.76 a | Lipid |
| Nonanal | 21.23 ±2.07 b | 27.15 ±2.86 a | 23.53 ±2.31 ab | Lipid |
| Citral | 6.99 ±0.49 ab | 7.97 ±0.47 a | 6.37 ±0.23 b | open chain carotenoid lycopene |
| 6-methyl-5-hepten-2-one | 216.62 ±22.13 b | 276.20 ±21.08 a | 305.52 ±18.41 a | open chain carotenoid lycopene |
| 6-methyl-5-hepten-2-ol | 10.77 ±2.04 a | 10.33 ±1.89 a | 11.94 ±1.55 a | open chain carotenoid lycopene |
| β-cyclocitral | 12.52 ±0.83 a | 11.34 ±1.01 a | 11.49 ±0.88 a | open chain carotenoid lycopene |
| geranial | 14.45 ±0.97 c | 17.45 ±0.45 b | 23.60 ±3.80 a | open chain carotenoid lycopene |
| geranylacetone | 54.47 ±4.97 a | 61.27 ±5.19 a | 66.48 ±6.18 a | open chain carotenoid lycopene |
| β-damascenone | 1.73 ±0.29 a | 1.97 ±0.03 a | 1.94 ±0.48 a | cyclic carotenoid lycopene |
| β-ionone | 6.27 ±0.34 a | 5.01 ±0.36 b | 4.66 ±0.52 b | cyclic carotenoid lycopene |
| 2-phenyl ethanol | 3.53 ±0.44 a | 2.12 ±0.19 b | 2.93 ±0.36 a | Phenylalanine |
| phenylacetaldehyde | 2.07 ±0.11 a | 2.24 ±0.29 a | 2.00 ±0.12 a | Phenylalanine |
| Benzyl alcohol | 2.12 ±0.27 a | 1.70 ±0.10 a | 2.18±0.17 a | Phenylpropanoid |
| benzaldehyde | 13.23 ±1.03 ab | 11.91± 0.64 b | 14.74 ±0.97 a | Phenylpropanoid |
| Eugenol | 3.02 ±0.41 a | 1.63 ±0.18 b | 2.07 ±0.26 b | Phenylpropanoid |
| 2-Octanone | 151.62 ±11.60 a | 138.89 ±8.91 a | 146.30 ±10.82 a | Branched chain amino acid |
| Butyl acetate | 23.69 ±2.53 a | 23.61±1.87 a | 22.88 ±1.04 a | Branched chain amino acid |
| 2-Isobutylthiazole | 64.13±8.51 b | 136.31±7.98 a | 109.65 ±17.49 a | Branched chain amino acid |

The values shown are means ± SD at R stage of four biological replicates (n=4). Means denoted by the same letter did not differ significantly according to ANOVA using Duncan test ((*P* <0.05).

**Supplementary Table 2. Specific primer sequences used for genetic transformation of *SlCYP90B3-OE* and *SlCYP90B3-RNAi* tomato plants.**

| Primer name | Primer sequence (5’- 3’) |
| --- | --- |
| *CYP90B3-OE-F* | ATGTCTGACTTAGAGTTTTTTCTTTTTCTT |
| *CYP90B3-OE-R* | GTCATGTAATTTATTTAACGATCGA |
| *CYP90B3-RNAi-F* | CCGCTCGAGTCTAGATTCTAGGTTGGGTTCTGA |
| *CYP90B3-RNAi-R* | CGGGGTACCAAGCTTCTGTGCTTCCTCCTTTAC |

| **Supplementary Table 3. Primer sequence of RT-qPCR** | | |
| --- | --- | --- |
| Primer name | Primer sequence（5’-3’） | Gene ID |
| *SlCYP90B3-F* | TGGAGGAGAGGCTTAAGGAA | *Solyc02g085360* |
| *SlCYP90B3-R* | GGCCAGCAAAGAGCAAACTC |  |
| *SlCPD-F* | CTTCTCTCCGAGCTGTTCATCTAG | *Solyc06g051750* |
| *SlCPD-R* | GAAGGAAAACAGAGAGTTCCACTC |  |
| *SlDWARF-F* | TCCTGATCCATATTCGTTCAA | *Solyc02g089160* |
| *SlDWARF-R* | ACCAAGTTCCTTTCCAGGAC |  |
| *SlCYP734A7-F* | CGGCAAACATACCACGTCTA | *Solyc03g120060* |
| *SlCYP734A7-R* | AGCCGCACAGATTCGTTAAT |  |
| *SlACS2-F* | CTACGCAGCCACTGTCTTTGAC | *Solyc01g095080* |
| *SlACS2-R* | TGATTCCGACTCTAAATCCTGGTAA |  |
| *SlACS4-F* | TTGCGACGAAATATATGCTGCT | *Solyc05g050010* |
| *SlACS4-R* | CACTCGAAATCCTGGAAAACCT |  |
| *SlACO1-F* | ACTATCCACCATGTCCTAAGCCCG | *Solyc07g049530* |
| *SlACO1-R* | TCTGTTTGTGCAATTACTCTGTGCAGC |  |
| *SlCTR1-F* | ACATTTGGATTATGTCAGGCTTGCA | *Solyc10g083610* |
| *SlCTR1-R* | TTGCTCAAACAATGGTTCAAAGAGG |  |
| *SlETR3-F* | AAGGGAACCACTGTCACGTTTGTAG | *Solyc09g075440* |
| *SlETR3-R* | TTAATGTTCTTTGTCACACCAATGTCC |  |
| *SlE4-F* | GACCACTCTAAATCGCCAGG | *Solyc03g111720* |
| *SlE4-R* | TTCCTGAGCGGTATTGCTTT |  |
| *SlE8-F* | TGGCTCCGAATCCTCCCAGTCT | *Solyc09g089580* |
| *SlE8-R* | GTCCGCCTCTGCCACTGAGC |  |
| *SlPG-F* | TCAAGGGCACAAGTGCAACAAAGG | *Solyc10g080210* |
| *SlPG-R* | TGCACGTAGCCTCTGATGGTTT |  |
| *SlDXS-F* | AGCTTCCGGCTGGAAACAAA | *Solyc01g067890* |
| *SlDXS-R* | CTAGCACAATAGCAGCATCC |  |
| *SlGGPPS-F* | GTACCTCGCTACCGCTACA | *Solyc04g079960* |
| *SlGGPPS-R* | TAATCCCACATTAGGGTTACC |  |
| *SlPSY1-F* | AACTTGTTGATGGCCCAAAC | *Solyc03g031860* |
| *SlPSY1-R* | CTGTATC GGACAAAGCACCA |  |
| *SlPDS-F* | TGGGTGGTTTGTCTACAGCAAA | *Solyc03g123760* |
| *SlPDS-R* | ATCCCTTGCCTCCAGCAGTA |  |
| *SlZDS-F* | TTGGAGCGTTCGAGGCAAT | *Solyc01g097810* |
| *SlZDS-R* | AGAAATCTGCATCTGGCGTATAGA |  |
| *SlCYC-B-F* | GTTCTGAAAGAAGTCATTCGGGTAATG | *Solyc04g040190* |
| *SlCYC-B-R* | CATGCCAATAACGAGGTTCTAAGTCA |  |
| *SlCCD1B-F* | AGAATCCAGATCTTGACGCGATT | *Solyc01g087260* |
| *SlCCD1B-R* | CCTCATCTCATACAACTCATTTGT |  |
| *ACTIN-F* | CCTCAGCACATTCCAGCAG | *Solyc03g078400* |
| *ACTIN-R* | CCACCAAACTTCTCCATCCC |  |

**Supplementary Fig. 1. Phylogenetic relationships among cytochrome P450s involved in brassinosteroid biosynthesis.** A neighbor-joining phylogenetic tree was constructed using the ClustalX software and deduced protein sequences.


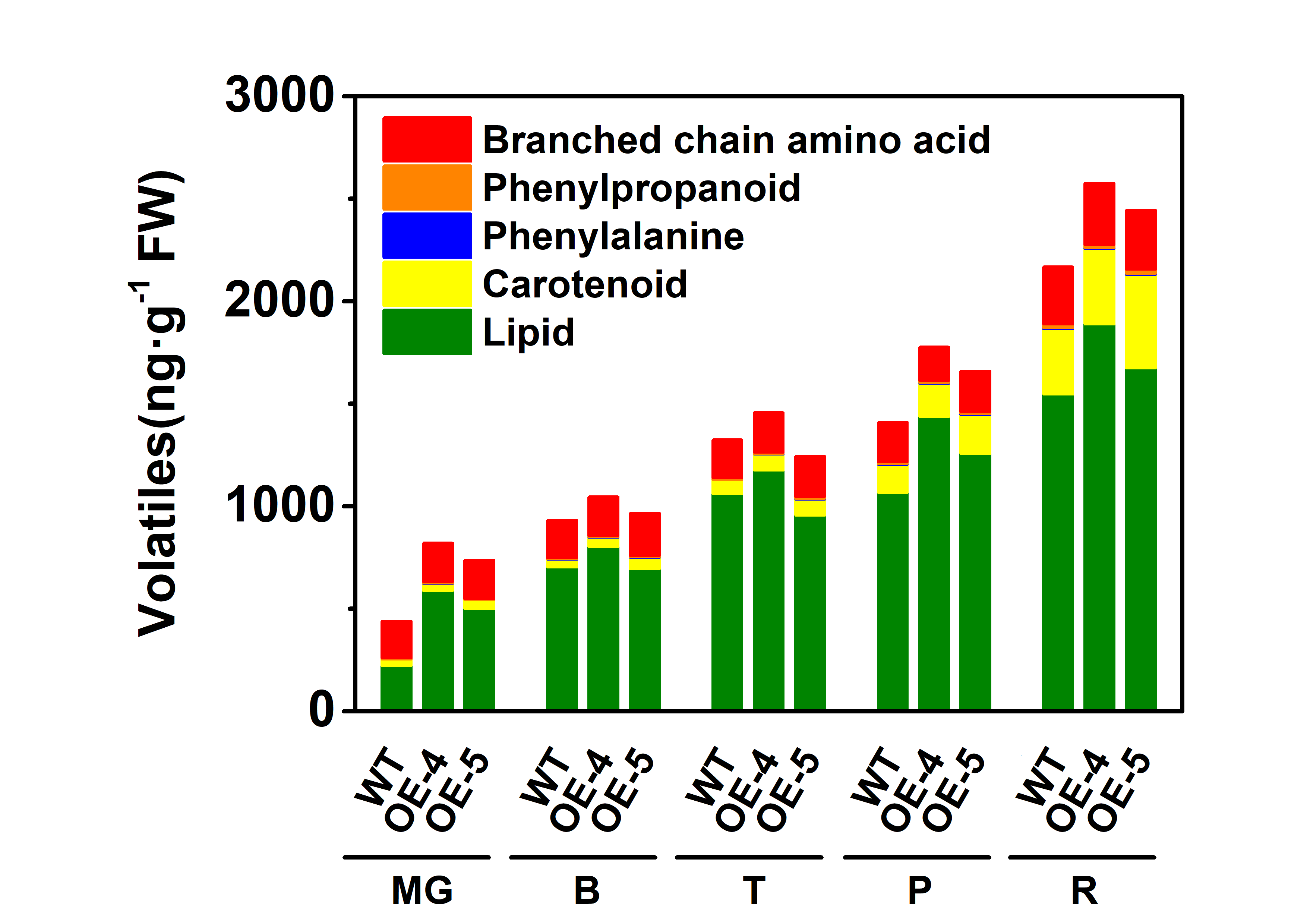


**Supplementary Fig. 2. Changes in volatiles derived from various pathways.** WT, wild type; MG, mature green; B, breaker; T, turning; P, pink; R, red.


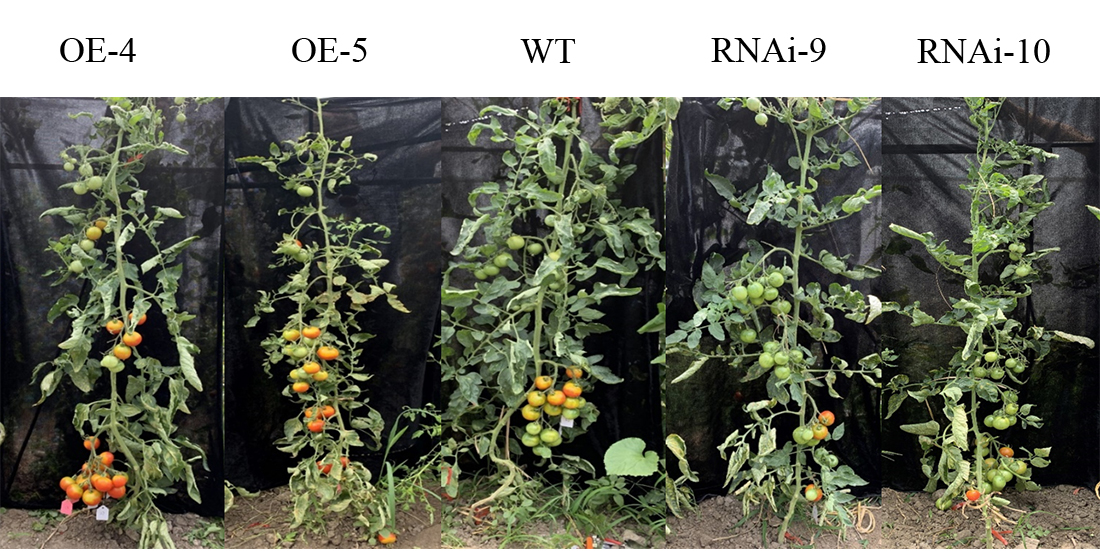


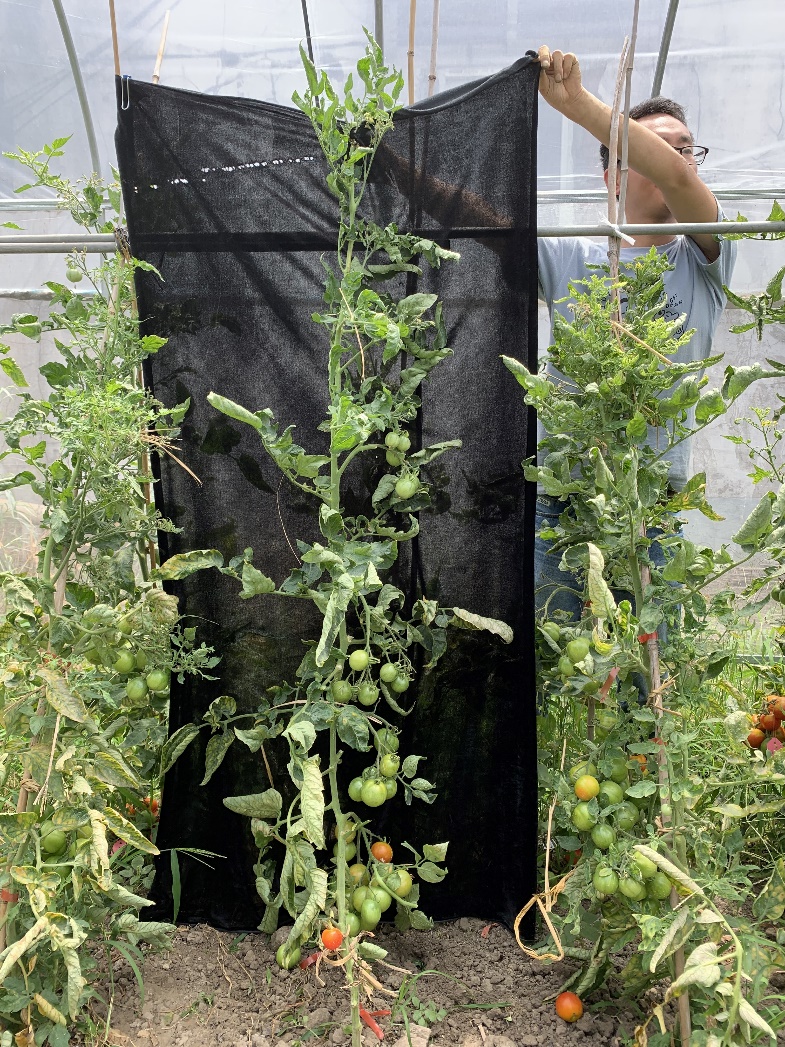


**Supplementary Fig. 3. Field phenotypes of WT and transgenic plants at 115 d after sowing.**
